# Wnt/β-catenin signaling and Msx1 promote outgrowth of the maxillary prominences

**DOI:** 10.3389/fphys.2012.00375

**Published:** 2012-09-21

**Authors:** Marie Medio, Erika Yeh, Antoine Popelut, Sylvie Babajko, Ariane Berdal, Jill A. Helms

**Affiliations:** ^1^Department of Orthodontics, Service of Odontology, Pitié-Salpêtrière Hospital, AP-HP, Paris 7 - Denis Diderot University, U.F.R. of OdontologyParis, France; ^2^Division of Plastic and Reconstructive Surgery, Department of Surgery, Stanford University School of MedicineStanford, CA, USA; ^3^Department of Periodontology, Service of Odontology, Rothschild Hospital, AP-HP, Paris 7 - Denis Diderot University, U.F.R. of OdontologyParis, France; ^4^Team Molecular Oral Pathophysiology, University Paris-Diderot, Centre de Recherche des Cordeliers – INSERM UMRS 872 - Université Paris Pierre et Marie Curie – INSERM UMRS 872 - Université Paris-DescartesFrance; ^5^Rare Disease Reference Center “Facial and Buccal Malformations”, Rothschild Hospital, AP-HPParis, France

**Keywords:** craniofacial, morphogenesis, ectopic, RCAS-Wnt2b

## Abstract

Facial morphogenesis requires a series of precisely orchestrated molecular events to promote the growth and fusion of the facial prominences. Cleft palate (CP) results from perturbations in this process. The transcriptional repressor Msx1 is a key participant in these molecular events, as demonstrated by the palatal clefting phenotype observed in *Msx1*^−/−^ embryos. Here, we exploited the high degree of conservation that exists in the gene regulatory networks that shape the faces of birds and mice, to gain a deeper understanding of Msx1 function in CP. Histomorphometric analyses indicated that facial development was disrupted as early as E12.5 in *Msx1*^−/−^ embryos, long before the palatal shelves have formed. By mapping the expression domain of *Msx1* in E11.5 and E12.5 embryos, we found the structures most affected by loss of Msx1 function were the maxillary prominences. Maxillary growth retardation was accompanied by perturbations in angiogenesis that preceded the CP phenotype. Experimental chick manipulations and *in vitro* assays showed that the regulation of *Msx1* expression by the Wnt/β-catenin pathway is highly specific. Our data in mice and chicks indicate a conserved role for Msx1 in regulating the outgrowth of the maxillary prominences, and underscore how imbalances in Msx1 function can lead of growth disruptions that manifest as CP.

## Introduction

Mid-facial morphogenesis involves the choreographed growth of the facial prominences with each other and with other regions of the growing head. When this highly synchronized process is disrupted, by either genetic or environmental influences, the result is cleft lip, cleft palate (CP), and the combination [CL/P; (Dixon et al., 2011)].

Avian and mammalian models have been used to study the molecular and cellular basis for CP and CL/P because during early embryonic development the faces of chicks, mice, and humans are remarkably similar (Helms and Schneider, 2003; Brugmann and Moody, 2005; Juriloff et al., 2005; Juriloff and Harris, 2008). Only during later stages of fetal development do species-specific facial characteristics emerge (Brugmann et al., 2006). This conservation in facial morphology is paralleled by an equally robust conservation in the gene regulatory networks that shape the face (Brugmann and Moody, 2005; Juriloff and Harris, 2008). Mouse models have an advantage in that like humans, slight variations in facial morphology can cause facial clefting (Juriloff et al., 2005, 2006; Juriloff and Harris, 2008). The disadvantage is that predisposing variations in facial form are difficult to detect (Boughner and Hallgrimsson, 2008; Boughner et al., 2008). Birds have much greater diversity in facial form, most noticeable when comparing different orders [i.e., ducks vs. quails (Helms and Schneider, 2003; Schneider and Helms, 2003)] but inter-species crosses are impossible. When used in combination, genetic models and experimental manipulations can provide useful insights into the molecular regulation of facial form.

We were interested in understanding more about the consequence of Msx1 deletion on facial development. Msx1 is a homeobox gene encoding transcriptional repressor and it functions in a variety of cell types to control processes as varied as proliferation, differentiation and angiogenesis (Marazzi et al., 1997; Odelberg et al., 2000; Hu et al., 2001; Han et al., 2003; Ishii et al., 2005; Lopes et al., 2011). The phenotype resulting from deletion of *Msx1* clearly demonstrates the critical role for this transcription factor plays in craniofacial development: *Msx1*^−/−^ mouse embryos have complete secondary CP, as well as tooth agenesis (Satokata and Maas, 1994). In humans, mutations in *MSX1* are among the genes implicated in CLP and CP (Lidral et al., 1998; Jezewski et al., 2003; Vieira et al., 2003; Suzuki et al., 2004; Tongkobpetch et al., 2006; Suazo et al., 2010; Salahshourifar et al., 2011).

Here, we made use of both mouse genetic models and avian approaches to gain a deeper appreciation of the role of Msx1 in facial development and facial clefting. Morphologic analyses of mice carrying null mutations in Msx1 revealed critical, early stages of prominence growth that were disrupted by loss of *Msx1*. Experimental manipulations in chick embryos underscored the critical importance of proper levels of Msx1 function for maxillary growth, and also highlighted the specific regulation of Msx1 by the Wnt/β-catenin pathway.

## Materials and methods

### Generation and genotyping of Msx1 embryos

All animal experiments were done in accordance with the Stanford University institutional guidelines. The Msx1 null allele was generated by the insertion of a n-LacZ insert in the second exon of mouse Msx1 gene (Houzelstein et al., 1997). Genotypes were confirmed by PCR using previously described primers and conditions (Houzelstein et al., 1997).

### Manipulation of chick embryos

Fertilized chicken eggs (*Gallus gallus*, Rhode Island Red Chickens from Petaluma Farms, Petaluma, CA) were prepared for surgical manipulations by making a small hole in the shell directly over the embryo. The replication competent retrovirus (RCAS) encoding *Wnt2b* (Cho and Cepko, 2006) was injected into the frontonasal prominence at St. 18. RCAS vectors are genetically programmed to infect cells and to integrate their genomes stably into host cells' DNA (Bell and Brickell, 1997); therefore, electroporation is not required.

### Collection and preparation of embryos

Mouse and chick embryos were collected in 4°C PBS then fixed in 4% paraformaldehyde (PFA) overnight at 4°C, dehydrated through an ethanol series and stored in 100% ethanol. Most tissues were embedded in paraffin and cut at 8 μm using a standard microtome. For each mouse stage we used an average of 7 embryos from the same litter, and embryos were collected at E12.5, E13.5, and E15.5. Chick embryos were collected at St. 17 HH, 20 HH, and 25 HH.

### Ethidium bromide staining and histomorphometry

**Figure 1:**
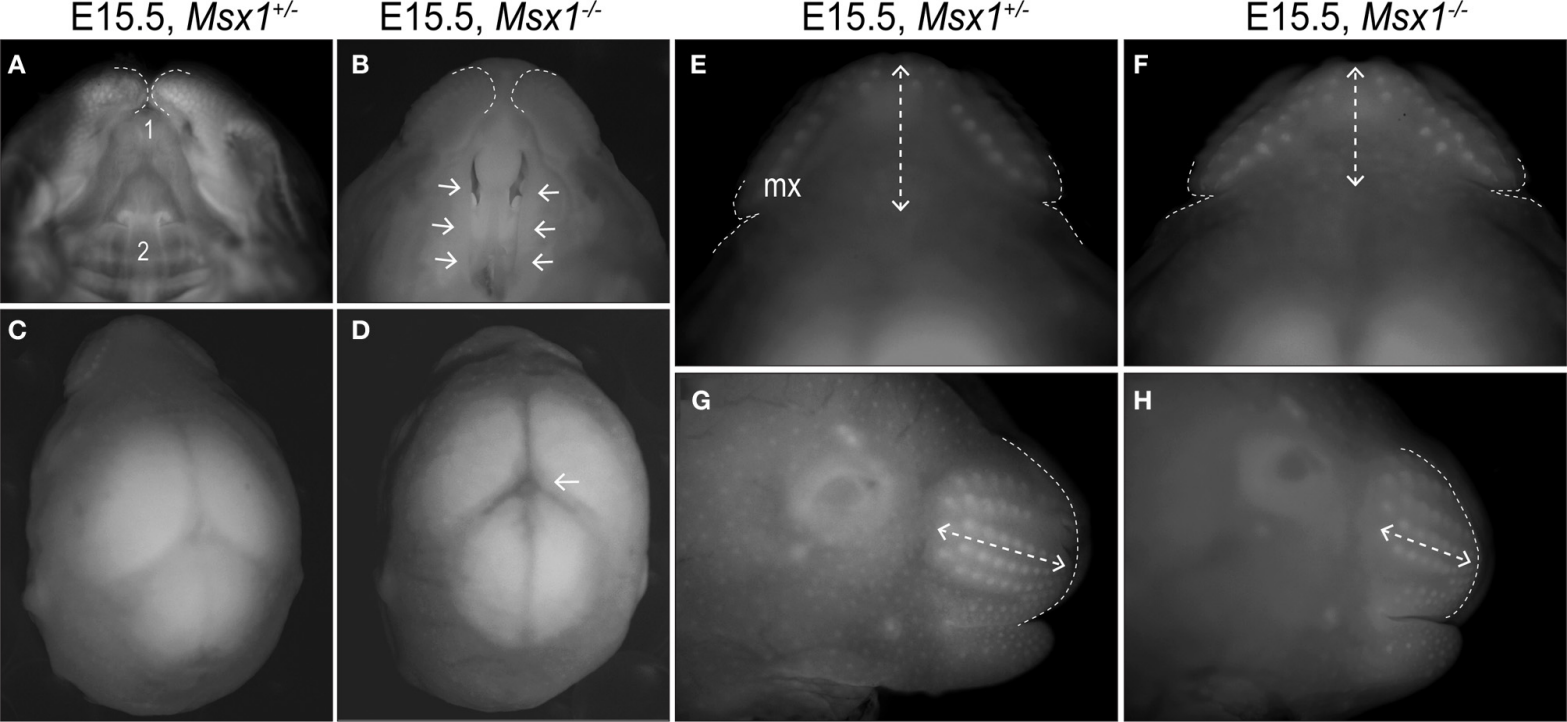
**Loss of Msx1 causes facial dysmorphologies in E15.5 mouse embryos. (A** and **B)** Ventral view of *Msx1*^+/−^ and *Msx1*^−/−^ embryos showing the primary (1) and secondary (2) palates. Dashed lines indicate the edges of the maxillary prominence. **(C** and **D)** Dorsal view of *Msx1*^+/−^ and *Msx1*^−/−^ embryos. Arrow points to abnormal organization of fiber tracts in the sub-commissural organ. **(E** and **F)** A higher magnification of *Msx1*^+/−^ and *Msx1*^−/−^ embryos; dashed lines indicates width of the rostrum (Maxillary width in Table 1). Dashed arrows indicate the length of the rostrum (maxillary length in Table 1). **(G** and **H)** Lateral view of *Msx1*^+/−^ and *Msx1*^−/−^ embryos. Dashed lines indicate outline of the maxillary prominence; dashed arrows indicate length of the rostrum. Mx, Maxillary.

**Figure 2:**
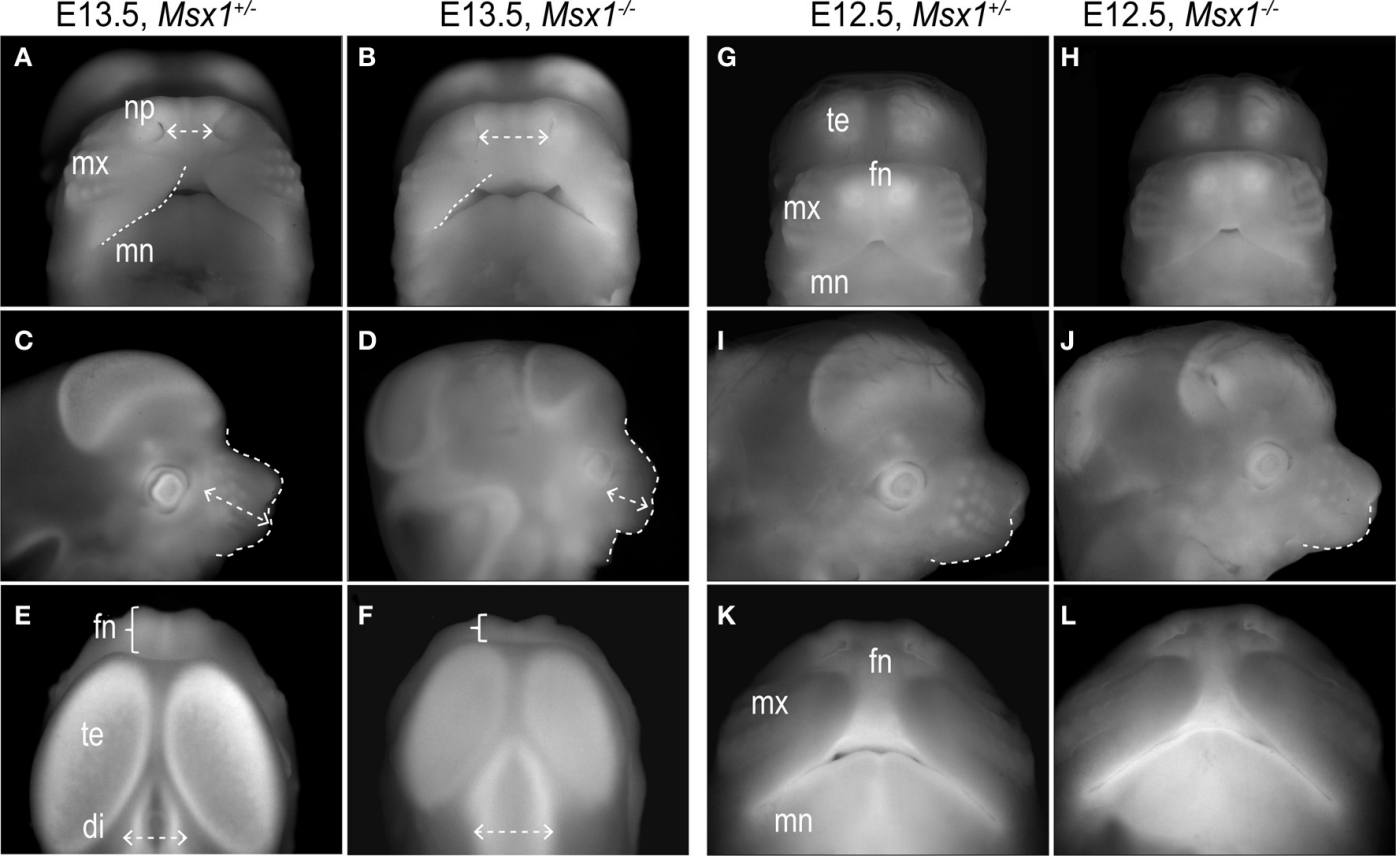
**Facial dysmorphologies in *Msx1*^−/−^ embryos are detectable at E13.5 but not at E12.5. (A** and **B)** Frontal view of E13.5 *Msx1*^+/−^ and *Msx1*^−/−^ embryos. Dashed lines indicate edges of the maxillary prominence and dashed arrows, distance between the nasal pits (internasal pit distance in Table 1). **(C** and **D)** Lateral view of *Msx1*^+/−^ and *Msx1*^−/−^ embryos. Dashed lines demarcate outline of the maxillary prominence; dashed arrow indicate length of the rostrum (maxillary length in Table 1). **(E** and **F)** Dorsal view of *Msx1*^+/−^ and *Msx1*^−/−^ embryos; bracket indicates size of the frontonasal prominence. Dashed arrows indicates width of the diencephalon. **(G** and **H)** Frontal view of E12.5 *Msx1*^+/−^ and *Msx1*^−/−^ embryos. **(I** and **J)** Lateral view, where dashed lines demarcates outline of the maxillary prominence. **(K** and **L)** Ventral view. di, diencephalon; fn, frontonasal prominence; mn, mandibular prominence; mx, maxillary prominence; np, nasal pit; te, telencephalon.

Mouse heads were incubated in 1xPBS containing ethidium bromide for 10 min and imaged in a 2× magnification using a dissecting microscope (Leica) under UV light. EtBr intercalates into DNA in cells of the epidermis and the resulting images, which are converted to grayscale (Figures 1, 2) clearly show surface topography.

Morphometric measurements were obtained from the EtBr images using the Adobe Photoshop “ruler” tool. Maxillary length: the largest distance between the nasal tip and the most proximal point of the maxilla from a lateral view; Maxillary width: the widest distance between the two extremes of the maxillary prominence from a frontal view; Internasal pit distance: the farthest distance between the nasal pit from a frontal view.

The measurements are expressed by mean and standard deviation, and the groups were compared by unpaired Student's *t*-test. A value *p* < 0.05 was considered statistically significant.

### Immunohistochemistry

For all antibody staining, de-paraffinized tissue sections were immersed in cold acetone and treated with 0.1% TritonX-100. Sections were incubated overnight at 4°C with a 1:1000 dilution of incubation in monoclonal anti-PECAM and anti-Col4a1 (Sigma, St. Louis, MO, USA) 1% donkey IgG overnight, and incubated for 1 h at room temperature in a 1:1000 dilution of FITC conjugated anti-mouse secondary antibody (Jackson Immunoresearch) in 1% donkey IgG. Slides were washed in 1:10 000 dilution of Hoechst in PBS. Image analysis of positive staining pixels was performed using Adobe Photoshop “color range” tool.

### *In situ* hybridization

Templates for the relevant mRNAs [mouse and chick *Msx1, gag* (Wang et al., 2004), *Wnt2b, Shh, Fgf8, Pax6* (Abzhanov et al., 2007)] were amplified from embryonic mouse cDNA by PCR using sequence-specific primers that included the promoter sites for T3 or T7 RNA polymerase. Antisense riboprobe for each gene was transcribed with either T3 or T7 RNA polymerase in the presence of Dig-11-UTP (mouse embryos; Roche; Indianapolis, IN, USA) or 35S-labeled riboprobes (chick embryos) (Albrecht et al., 1997). Hybridizations and washes were performed at high stringency as described in detail elsewhere (Albrecht et al., 1997).

Images of radioactive *in situ* hybridization assays are pseudo-colored superimpositions of the *in situ* hybridization signal and a blue nuclear stain (Hoescht Stain, Sigma) that are made using Adobe Photoshop. Briefly, two separate images were captured in Adobe Photoshop. One image was a fluorescent image of the nuclei, and the other was a dark field image of the *in situ* hybridization signal. These images are superimposed as different layers within Photoshop. The “colorize” tool is used to add a contrasting color to the *in situ* hybridization layer. No changes in threshold intensities are made; however, slight adjustments to the contrast and brightness were performed to accurately reflect what is observed with the microscope. The images are then flattened for importation into Adobe Illustrator, where the final figures were assembled.

### Mouse embryonic fibroblast (MEF) treatment with Wnt ligands

Wild-type mouse embryos (*n* = 3) were harvested at stage E11.5. Their limb buds were mechanically disrupted with a pipette and they were cultured in 25 cm^2^ flasks for 48 h in DMEM-High Glucose (Gibco) supplemented with 10% fetal bovine serum (Gibco) and 1% penicillin/streptomycin (Gibco). Once attached, cells were passaged into 6-well plates, 3 wells per condition. The same volume of either phosphate buffered saline, Wnt3a (0.2 ng/ml; R&D Systems) or Wnt5a (0.2 ng/ml; R&D Systems) was added into each well. After 4 h, RNA was extracted with RNeasy mini kit (Qiagen). cDNA was prepared from 1 μg of each RNA sample with SuperScript III kit (Invitrogen). Quantitative real time PCR for *Msx1* (Forward primer: ATGCTCTGGTGAAGGCCGAAAG; reverse primer: TTGCGGTTGGTCTTGTGCTTGC) and *Gapdh* (Forward primer: CATGGCCTTCCGTGTTCCTA, reverse primer: GCGGCACGTCAGATCCA) was performed with Sybr Green mix in ABI 7900HT system (Applied Biosystems).

## Results

### Craniofacial abnormalities and cleft palate in Msx1^−/−^ mouse development

Analyses of E15.5 *Msx1*^−/−^ embryos confirmed the previously described phenotype of clefting in the secondary palate [Figures 1A,B and see (Satokata and Maas, 1994; Hu et al., 2001; Zhang et al., 2002; Levi et al., 2006)]. We also observed the abnormal organization of fiber tracts in the sub-commissural organ (Figures 1C,D) as described previously (Bach et al., 2003; Ramos et al., 2004).

*Msx1*^−/−^ embryos are described as having a “deficiency in both mandibular and maxillary development” (Satokata and Maas, 1994); we focused on this aspect of craniofacial development. E15.5, *Msx1*^−/−^ embryos have a rostrum that is 28% shorter along the coronal plane (*p* = 0.002) but not appreciably wider along the sagittal plane (*p* = 0.09, Table 1; Figures 1E,F). From a dorsal perspective, this foreshortening of the rostrum led to the appearance of more whisker primordia in *Msx1*^−/−^ embryos. From a lateral perspective, however, it was evident that the number of whisker primordia was equivalent but their distribution was changed: *Msx1*^−/−^ embryos had more tightly packed whisker primordia compared to heterozygous littermates (Figures 1G,H). *Msx1*^−/−^ embryos did, however, have fewer hair follicle primordia covering the lateral sides of their developing faces (Figures 1G,H).

**Table 1:** **Histomorphometric comparison of the heads from *Msx1*^+/−^ and *Msx1*^−/−^ embryos (E12.5, E13.5, and E15.5)**.

**Measurement**	**Embryo stage**	**Length ± st.dev. (mm)**		***p*-value (unpaired *t*-test)**
		***Msx1*^+/−^**	***Msx1*^−/−^**	
Maxillary length	E15.5	19.0 ± 1.8 (*n* = 7)	14.8 ± 1.1 (*n* = 5)	0.002^*^
Maxillary width	E15.5	34.2 ± 3.4 (*n* = 7)	30.8 ± 2.9 (*n* = 5)	0.09 (n.s.)
Internasal pit distance	E13.5	5.6 ± 0.4 (*n* = 11)	7.7 ± 0.5 (*n* = 7)	<0.0001^*^
Maxillary length	E13.5	8.1 ± 0.7 (*n* = 11)	5.2 ± 0.5 (*n* = 7)	<0.0001^*^
Internasal pit distance	E12.5	7.5 ± 0.8 (*n* = 4)	8.2 ± 0.5 (*n* = 5)	0.2 (n.s.)

Fiducials used to make the measurements are shown in Figures 1, 2.

Maxillary length, the largest distance between the nasal tip and the most proximal point of the maxilla from a lateral view; Maxillary width, the widest distance between the two extremes of the maxillary prominence from a frontal view; Internasal pit distance, the farthest distance between the nasal pit from a frontal view.

* *p* < 0.05.

In order to see when these facial alterations are first observed, we collected embryos at progressively earlier stages and undertook similar anatomical comparisons between homozygous embryos and their heterozygous or wild-type littermates (Table 1). At E13.5, *Msx1*^−/−^ embryos had a wider mid-face, as shown by the 37% increase in the distance between the nasal pits (*p* < 0.0001, Table 1; Figures 2A,B). The *Msx1*^−/−^ maxillary prominences were shorter in the transverse plane (dotted white line) and because of this the nasal cavity was visible from a frontal view (Figures 2A,B). From a lateral perspective, *Msx1*^−/−^ embryos also displayed a 55% shortened rostrum in keeping with the phenotype of E15.5 embryos (*p* < 0.0001, Table 1; Figures 2C,D). From a superior or dorsal perspective, the foreshortened rostrum was clearly evident (brackets; Figures 2E,F). The telencephalic vesicles of the *Msx1*^−/−^ embryos were also reduced along the coronal plane and the diencephalon was wider in the sagittal plane (Figures 2E,F) in accordance with the role of Msx1 in patterning the midbrain (Bach et al., 2003; Ramos et al., 2004).

At E12.5, frontonasal, maxillary, and mandibular prominences of *Msx1*^−/−^ embryos appeared equivalent in size, shape, and position relative to littermate controls (Table 1; Figures 2G,H). From a lateral perspective, a very slight, but not significant shortening of the maxillary prominences was observed in the mutant (Table 1; Figures 2I,J). From an inferior or ventral perspective, this subtle difference was not apparent (Figures 2K,L).

From these histomorphometric analyses we conclude that the developmental events leading to the CP observed in *Msx1*^−/−^ embryos are initiated between E12.5 and E13.5. This conclusion is in contrast to other studies that have attributed the *Msx1*^−/−^ clefting phenotype to a failure of the palatal shelves to grow and to fuse (Hu et al., 2001; Zhang et al., 2002; Levi et al., 2006). Our data suggest that earlier developmental events contribute to the facial clefting defect, although we cannot rule out that a compounded phenomenon between maxillary prominence and palatal shelves growth failure might be causing CP. Our next analyses focused on identifying these Msx1-dependent developmental events.

### Evaluating Msx1 expression in craniofacial development

**Figure 3:**
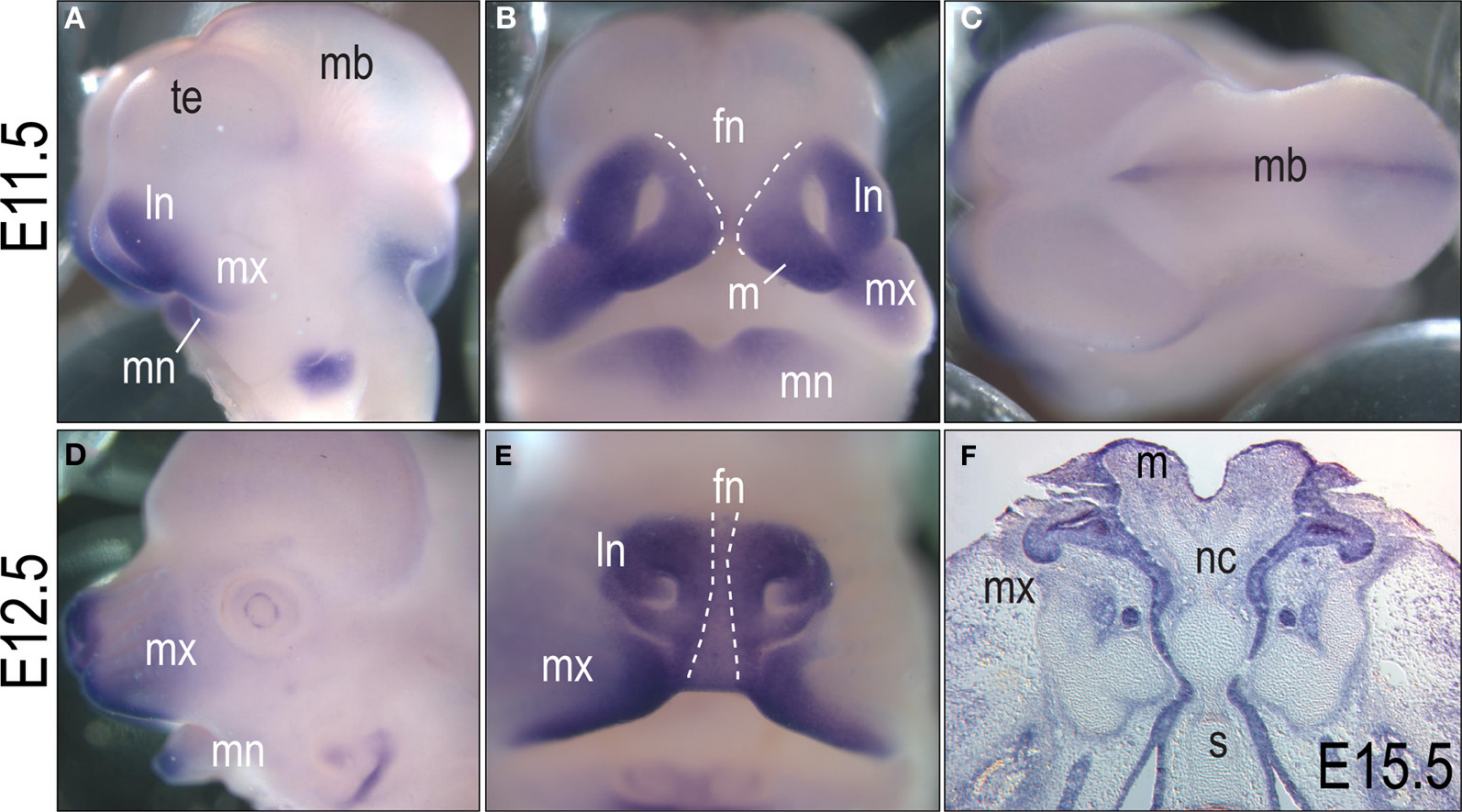
***In situ* hybridization for *Msx1* at E11.5, E12.5 and E15.5 in the developing face. (A)** Lateral view of an E11.5 embryo shows Msx1 expression in the maxillary, mandibular, and lateral nasal prominences and in the dorsal portion of the midbrain. **(B)** Frontal view illustrating that the midline of frontonasal prominence (dashed lines) lacks *Msx1*. **(C)** Dorsal view showing *Msx1* expression in the midline of the midbrain. **(D)** Lateral view at E12.5; *Msx1* transcripts are detectable in the maxillary and mandibular prominences. **(E)** Frontal view of the developing snout in an E12.5 embryo. The midline of frontonasal prominence (dashed lines) lacks *Msx1* expression. **(F)** Coronal section of an E15.5 embryo. *Msx1* is expressed in the epithelium of the maxillary and mandibular prominences. fn, frontonasal prominence; ln, lateral nasal prominence; m, medial nasal prominence; mb, midbrain; mn, mandibular prominence; mx, maxillary prominence; np, nasal pit; te, telencephalon.

In order to understand, which developmental events might be altered in *Msx1*^−/−^ embryos between E12.5 to E13.5, we evaluated *Msx1* gene expression in the developing face [see also (Mackenzie et al., 1991; Satokata and Maas, 1994; Zhang et al., 2002; Levi et al., 2006)]. At E11.5, *in situ* hybridization analyses in wild-type embryos showed expression of *Msx1* in the maxillary, lateral nasal, and mandibular prominences, as well as the second pharyngeal arch and otic capsule region (Figure 3A). From a frontal view, *Msx1* was expressed in the maxillary, lateral nasal, and fusing portions of the mandibular prominences but was conspicuously absent from the frontonasal midline (Figure 3B). From a superior view, *Msx1* was expressed in the midline of the developing cranial vault (Figure 3C). Note, however, that this mesenchymal expression domain precedes any ossification of the skull bones by almost 3.5 embryonic days.

At E12.5, the expression domain of *Msx1* was more restricted to growing edges of the maxillary and mandibular prominences (Figures 3D,E). As observed at earlier stages, *Msx1* transcripts are less abundant in the frontonasal midline (dotted lines, Figure 3E). At E15.5, Msx1 was primarily expressed in the whisker primordia and in the epithelium of the maxillary and mandibular prominences (Figure 3F). From these molecular analyses we concluded that the sites most likely affected by loss of Msx1 function were the maxillary, median and lateral nasal prominences. Given the continued expression of *Msx1* in the growing edges of the maxillae, and the phenotypes of *Msx1*^−/−^ embryos, we focused our attention on this prominence and its derivatives.

### Msx1 regulates angiogenesis in the developing face

**Figure 4:**
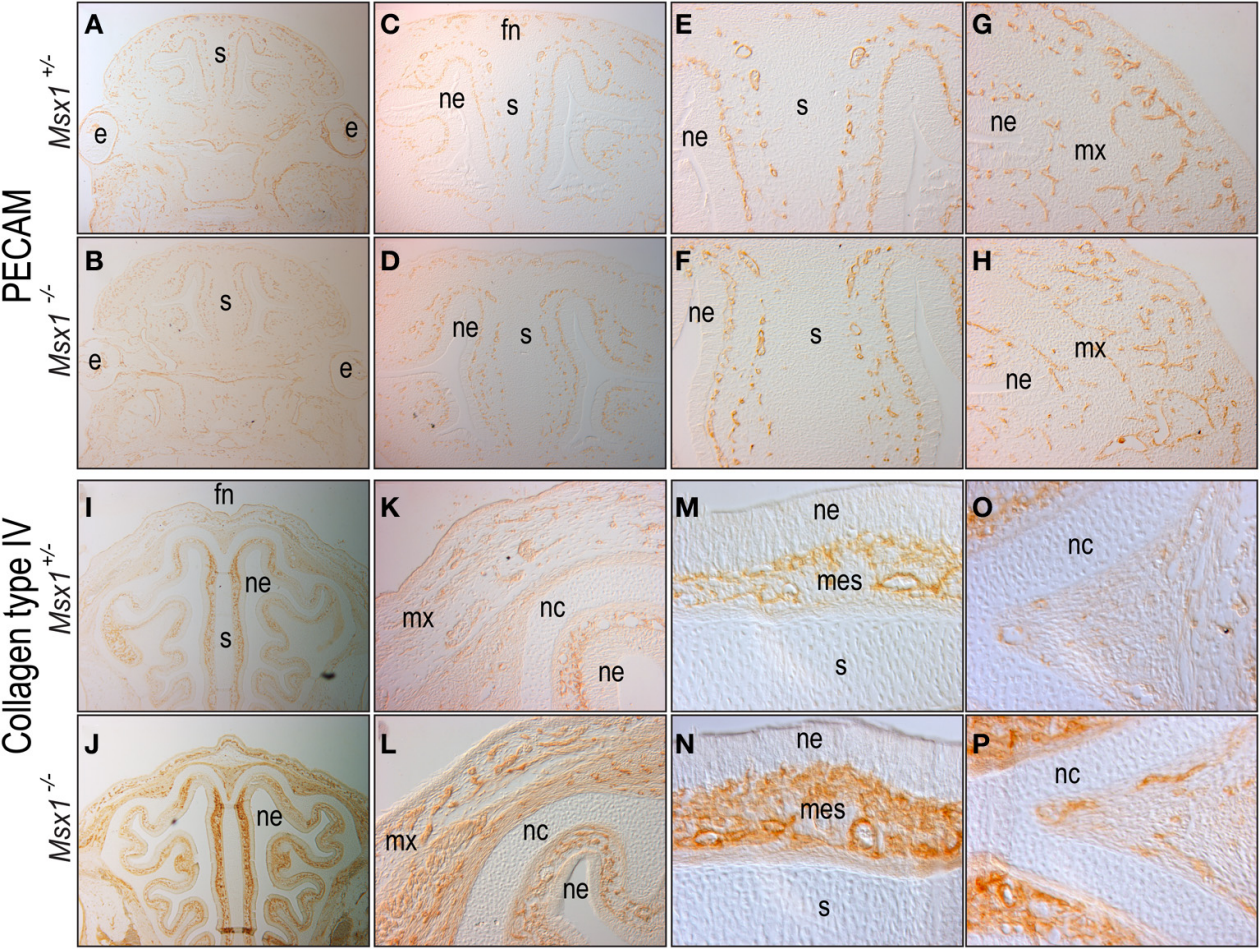
**Loss of *Msx1* leads to abnormal angiogenesis.** Immunostaining for the endothelial marker PECAM in coronal sections of E12.5 **(A,C,E,G)** *Msx1*^+/−^ and **(B,D,F,H)** *Msx1*^−/−^ embryos in increasing magnifications: 2.5× **(A,B)**, 5× **(C,D)**, detailed frontonasal prominence at 10× **(E,F)**, and detailed maxillary prominence at 10× **(G,H)**. Immunostaining for the pericyte marker Type IV collagen in coronal sections of E15.5 **(I,K,M,O)** *Msx1*^+/−^ and **(J,L,N,P)** *Msx1*^−/−^ embryos in increasing magnifications: 5× **(I,J)**, 10× **(K,L)**, detailed nasal septum at 20× **(M,N)**, and detailed nasal cartilage at 20× **(O,P)**. e, eye; fn, frontonasal prominence; mes, mesenchyme; mx, maxillary prominence; nc, nasal cartilage; ne, nasal ectoderm; s, nasal septum.

In order to understand how ablation of *Msx1* expression affected growth of the maxillary prominences, we prepared matching tissue sections from control *Msx1*^+/−^ and *Msx1*^−/−^ embryos and monitored how vascularization was affected by loss of *Msx1*. Using platelet endothelial cell adhesion molecule (PECAM) to identify endothelial cells (Albelda et al., 1991) we first evaluated embryos for changes in the pattern of angiogenesis. Our reasoning was that the foreshortened maxillary prominences in the *Msx1*^−/−^ embryos might result from a lack of oxygen and nutrients delivered via the bloodstream. In E12.5 *Msx1*^+/−^ controls, PECAM immunostaining of blood vessels was broadly distributed throughout the maxillary and frontonasal mesenchyme (Figures 4A,C). Image analyses of *Msx1*^+/−^ embryos revealed that 0.9% of the pixels in the maxillary prominence were positive for PECAM staining (Figures 4E,G). We also noted that the lumen of the vessels was uniformly small (Figures 4E,G). We observed a similar distribution of blood vessels in *Msx1*^−/−^ embryos (Figures 4B,D) with one notable exception: the lumens of the vessels were on average much larger (Figures 4F,H), which altered their density. Using image analyses we found that in *Msx1*^−/−^ embryos only 0.3% of the pixels in the same region were PECAM positive (compare Figures 4E,F).

To confirm the vascular phenotype in *Msx1*^−/−^ embryos, we made use of Type IV collagen immunostaining to identify pericytes that surround mature blood vessels (Jeon et al., 1996). In E15.5 *Msx1*^+/−^ embryos, ColIV staining was evident throughout the facial mesenchyme and nasal septum (Figures 4I,K), with a stronger staining near the nasal cartilage and between the nasal cartilage and the nasal ectoderm (Figures 4M,O). In *Msx1*^−/−^ embryos there was a clear enrichment in pericytes-covered blood vessels next to the nasal cartilage and nasal ectoderm (Figures 4J,L). Image analyses indicated there was a 1.3-fold enrichment in ColIV staining in *Msx1*^−/−^ embryos (Figures 4N,P), suggesting that the earlier vascular defect had undergone a rebound, resulting in a greater density of blood vessels in the E15.5 *Msx1*^−/−^ face.

### Msx1 is regulated by Wnt/β-catenin signaling

**Figure 5:**
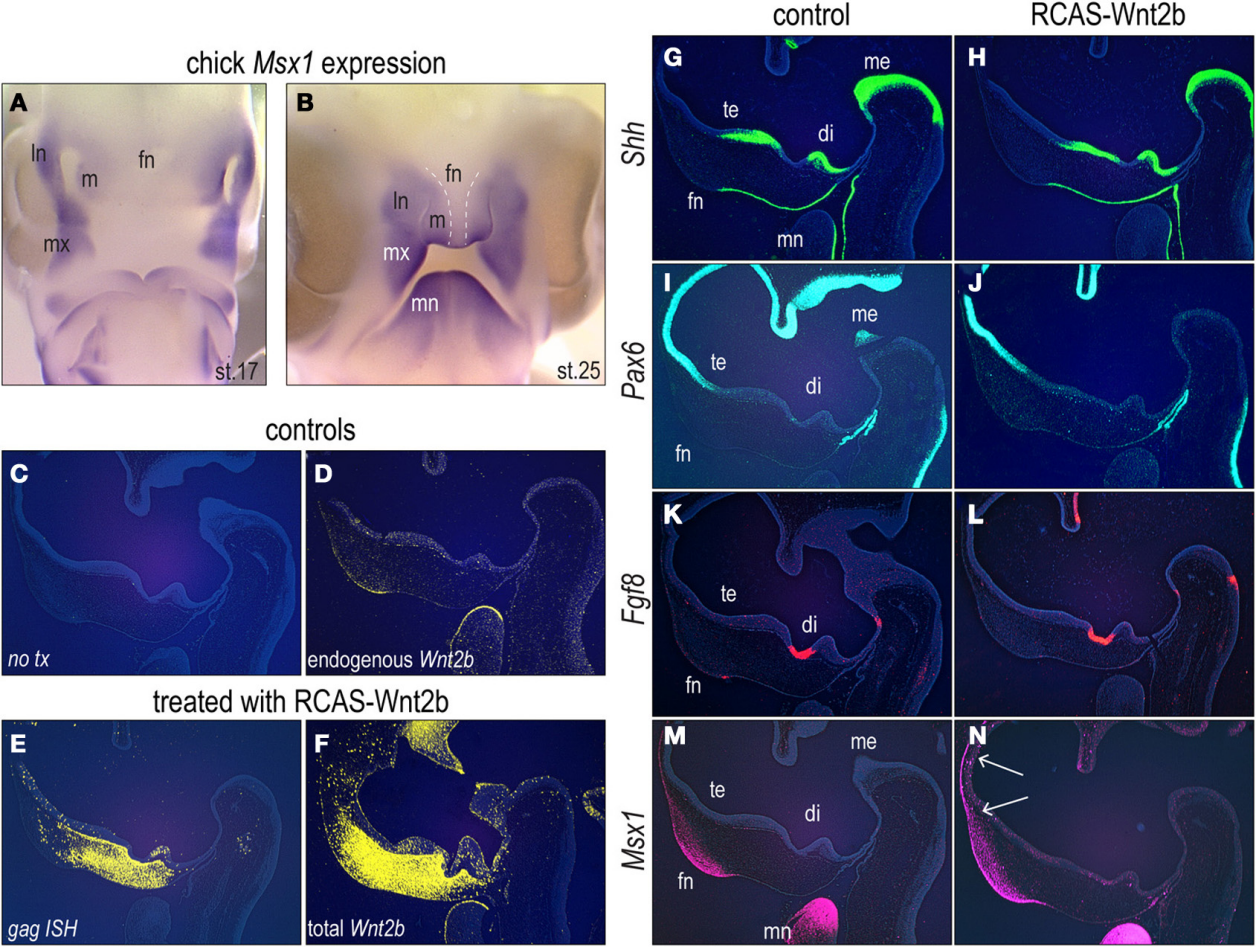
***Wnt2b* over-expression leads to ectopic *Msx1* in the facial prominences. (A)** *In situ* hybridization for *Msx1* in the avian facial prominences at stage 17 HH. **(B)** *In situ* hybridization for *Msx1* in the avian facial prominences at stage 25 HH. Dashed lines delimit the lack of *Msx1* expression in the midline of frontonasal prominence. **(C,D)** Mid-sagittal section through the head of an uninjected embryo. **(D)** Endogenous Wnt2b expression in the same embryo. **(E)** Mid-sagittal section through the head of an embryo, analysed 24 h after *RCAS:Wnt2b* infection, showing viral delivery to the face. **(F)** *RCAS:Wnt2b* infection drives ectopic *Wnt2b* expression. Forty-eight hours after *RCAS:Wnt2b* treatment, chicken embryos were assessed for alterations in **(G,H)**, *Shh*, **(I,J)** *Pax6*, **(K,L)** *Fgf8* and **(M,N)** *Msx1* expression. White arrows indicate expanded *Msx1* expression domain. Di, diencephalon; fn, frontonasal prominence; ln, lateral nasal prominence; m, medial nasal prominence; mb, midbrain; me, metencephalon; mn, mandibular prominence; mx, maxillary prominence; np, nasal pit; te, telencephalon.

Msx1 orthologs are found in a variety of species, from *C. elegans* to vertebrates including *Gallus gallus*, *Mus musculus*, and *Homo sapiens*. Given the highly conserved nature of the DNA sequence we wondered if the pattern of gene expression, and thus perhaps the function of Msx1, was conserved among these species. We chose an avian model because of the ease with which embryos can be manipulated, collected, and evaluated [Figures 5A,B; and see Brugmann et al. (2010)]. At stage 17 HH, *Msx1* was strongly expressed in the medial and lateral edges of the nasal pits, throughout the maxillary prominences and in the distal and proximal edges of the mandibular prominences (Figure 5A). At stage 25 HH, *Msx1* expression was observed mainly in the mandibular and maxillary prominences (Figure 5B). It was also expressed in the lateral nasal and medial nasal prominences, but was excluded from the midline (frontonasal) portion (Figure 5B). These expression domains are highly reminescent to those in the developing mouse face (Figure 3).

Others (Song et al., 2009) and we hypothesize that Wnt/β-catenin signaling regulates *Msx1* transcription. To test this theory we over-expressed a Wnt ligand in the developing facial prominences of chick embryos and then assessed how this over-activation of the Wnt pathway affected *Msx1* expression. Chicken embryos at stage 18 HH were injected with *RCAS:Wnt2b* (Cho et al., 2006). We evaluated morphology (Figure 5C), and endogenous pattern of *Wnt2b* and found transcripts in the frontonasal ectoderm and in the mandibular ectoderm (Figure 5D).

Twenty-four hours after delivery of *RCAS:Wnt2b*, we used *in situ* hybridization to assay for the spread of the virus using *in situ* hybridization for *gag* [(Wang et al., 2004) and see Figure 5E], and for the ectopic (and endogenous) expression of *Wnt2b* (Figure 5F). *Wnt2b* was broadly expressed throughout the frontonasal mesenchyme, up to the metencephalon (Figure 5F). It is possible that, as a growth factor, Wnt2b signals to cells further from infected areas, increasing *Wnt2b* expression.

We evaluated how ectopic *Wnt2b* expression affected the expression domains of other molecular markers in the facial prominences analyzing consecutive sections. We focused on three key regulators: Shh (Cordero et al., 2004; Marcucio et al., 2005; Abzhanov et al., 2007), Pax6 (Goudreau et al., 2002), and Fgf8 (Schneider et al., 2001) because of their pivotal roles in facial development. Forty-eight hours after *RCAS:Wnt2b* delivery, the *Shh* expression domains in the ventral portions of the telencephalon, diencephalon and metencephalon, as well as in the facial midline, were equivalent between control and *RCAS:Wnt2b* treated embryos (Figures 5G,H).

Shh represses *Pax6* expression (Macdonald et al., 1995), and as expected, the pattern of *Pax6* expression was precisely opposite of the *Shh* expression domains: *Pax6* transcripts were detected in the dorsal regions of the telencephalon and metencephalon and in the Rathke's pouch (Figures 5I,J). As we had observed with the *Shh* analyzes, *Pax6* expression domains were also unchanged by *RCAS:Wnt2b* treatment (Figures 5I,J). *Fgf8* expression domains in the diencephalon and the frontonasal ectoderm were also similar between control and *RCAS:Wnt2b* treated embryos (Figures 5K,L).

In sharp contrast, *Msx1* expression domains were altered by *RCAS:Wnt2b*. Endogenous *Msx1* was expressed in the ectoderm and surrounding mesenchyme of the anterior portion of the frontonasal prominence as well as in the mandibular prominence (Figure 5M). In *RCAS:Wnt2b* treated embryos, the *Msx1* expression domain was expanded dorsally, in both the facial ectoderm and mesenchyme, as well as in Rathke's pouch at the 48-h time point (Figure 5N). This ectopic *Msx1* domain (Figure 5N) was in the same general vicinity as the ectopic expression of Wnt2b (Figure 5F).

These data showed that over-expression of *Wnt2b* did not affect the domains of *Shh*, *Pax6* or *Fgf8*. Ectopic Wnt activation in the face, however, altered the expression of *Msx1.* Thus, we conclude that Wnt signals regulate *Msx1* expression in the embryonic face.

### Wnt signaling and Msx1 regulate development of the maxillary prominences

**Figure 6:**
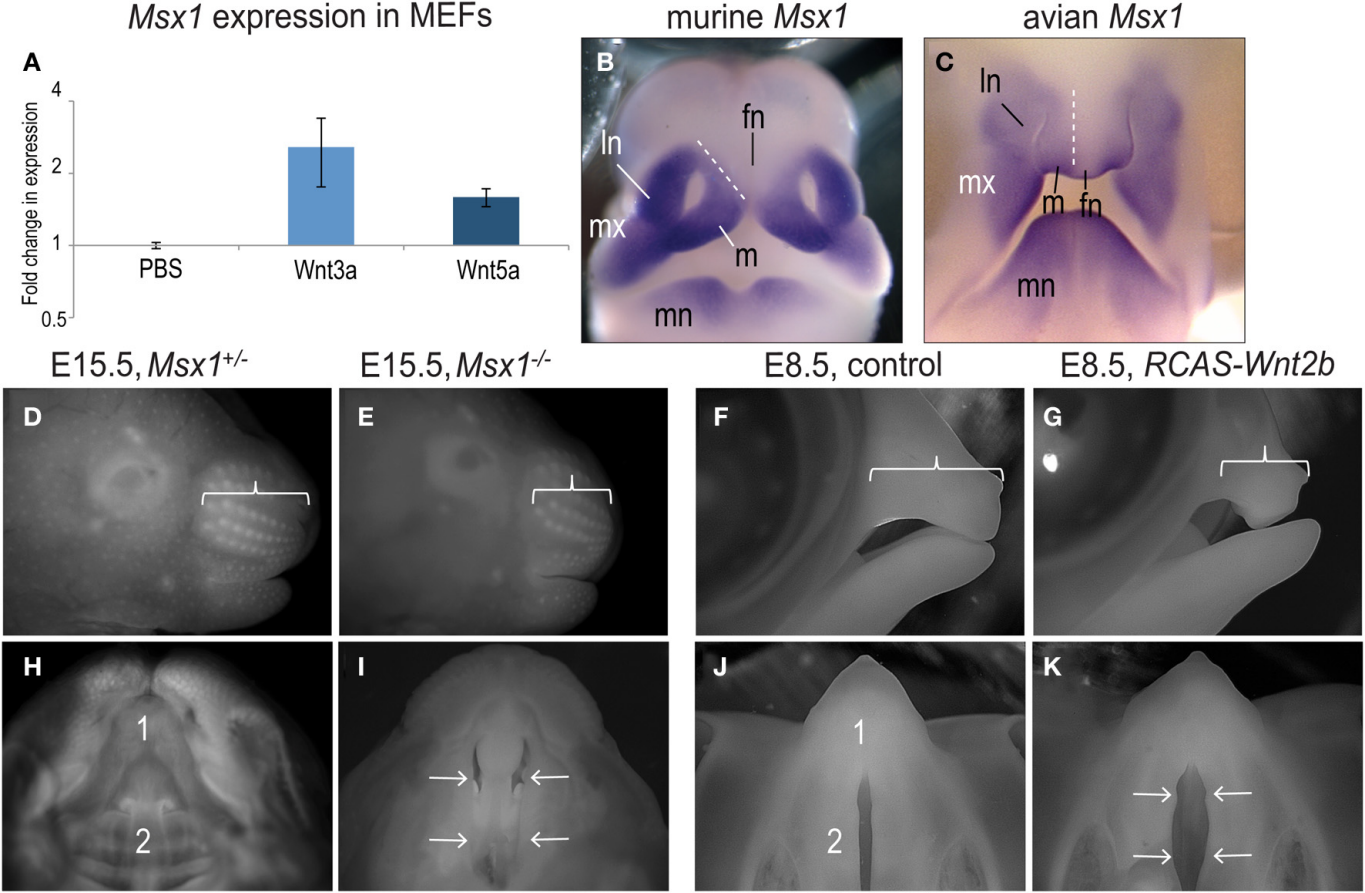
**Loss and gain of *Msx1* expression leads to a similar clefting phenotype. (A)** *Msx1* gene expression induced by Wnt3a or Wnt5a treatment in E11.5 MEFs. **(B)** Comparison between *in situ* hybridization of *Msx1* in mice and **(C)** chick embryos. **(D)** Lateral view of E15.5 *Msx1*^+/−^ and **(E)** *Msx1*^−/−^ embryos. Bracket indicates the length of the rostrum. **(F)** Lateral view of control and **(G)** *RCAS-Wnt2b* treated E8.5 chicken embryos. Bracket indicates the length of the upper beak. **(H)** Ventral view of E15.5 *Msx1*^+/−^ and **(I)** *Msx1*^−/−^ embryos. Primary (1) and secondary (2) palates are fused in *Msx1*^+/−^ embryos but the secondary palate is cleft in *Msx1*^−/−^ embryos (arrows). **(J)** Ventral view of control and **(K)** *RCAS:Wnt2b* treated E8.5 chicken embryos. Primary (1) palate is fused while secondary (2) palate is naturally cleft in control embryos; note widening of secondary palate secondary to ectopic *Msx1* expression (arrows). fn, frontonasal prominence; ln, lateral nasal prominence; m, medial nasal prominence; mn, mandibular prominence; mx, maxillary prominence.

In the developing chick, Wnt signaling regulates *Msx1* expression in the facial prominences. To confirm this regulatory role, we isolated mouse embryonic fibroblasts from E11.5 embryos and exposed the cells to a Wnt stimulus for 4 h. Quantitative RT-PCR for *Msx1* and *Gapdh* demonstrated that Wnt3a significantly enhanced expression of *Msx1* (Figure 6A). Cells were also treated with Wnt5a, which has recently been shown to activate both beta catenin-dependent and independent Wnt signaling (van Amerongen et al., 2012), and again we found that *Msx1* expression was significantly increased (Figure 6A). Collectively, these molecular analyses verify that Wnt stimuli can directly activate *Msx1* gene transcription.

We returned to evaluated the *RCAS-Wnt2b* treated embryos and evaluated their facial phenotypes. Based on their analogous expression domains in mouse (Figure 6B) and chick embryos (Figure 6C), we were surprised to find that ectopic expression of *Msx1* in chicks produced a clefting phenotype, very similar to the phenotype resulting from loss of *Msx1* in mice. For example, loss of *Msx1* leads to a foreshortened maxilla in early mouse embryos (Figures 6D,E) and in chick embryos, ectopic *Msx1* expression led to a similar foreshortened rostrum/upper beak (Figures 6F,G). In mouse embryos, the foreshortened rostrum leads to a clefting phenotype (compare Figure 6H with 6I). Birds have a naturally occurring “cleft” between the palatal shelves, but in chick embryos over-expressing *Msx1* the palatal shelves were separated by a much larger distance (compare Figure 6J with 6K). These data indicate that disturbances in the balance of *Msx1* expression interrupt maxillary prominence outgrowth, which contributes to palatal clefts.

## Discussion

### Loss of Msx1 regulates growth of the maxillary prominences

Though its interactions with a core transcription complex and other homeobox containing genes, *Msx1* functions as a transcriptional repressor (Alappat et al., 2003). In the developing face, its primary site of activity is in the growing edges of the lateral nasal, median nasal and maxillary prominences (Figures 3, 6). At later stages of development, *Msx1* is restricted to the anterior part of the palatal shelves (Zhang et al., 2002; Hilliard et al., 2005) and *Msx1*^−/−^ embryos show complete secondary cleft palate (Satokata and Maas, 1994).

The cleft palate phenotype has been attributed to cell proliferation defects in the anterior region of the developing palatal shelves at E13.5 (Hu et al., 2001; Zhang et al., 2002; Levi et al., 2006) but our analyses demonstrate that loss of *Msx1* disturbs facial developmental events as early as E12.5 (Figures 1, 2). The *Msx1* gene expression domains at E11.5 and E12.5, together with anatomical analyses, pinpoint that the maxillary prominence is one of the primary facial structure affected by ablation of *Msx1.* These data demonstrate that the *Msx1*^−/−^ palatal phenotype develops as a compounded phenomenon to a disruption in the outgrowth of the maxillary prominences. In other facial regions *Msx1* controls cell proliferation (Zhang et al., 2002; Han et al., 2003), angiogenesis (Lopes et al., 2011) and tissue patterning (Coudert et al., 2005; Ishii et al., 2005). We find that angiogenesis is disrupted in the *Msx1*^−/−^ maxillary prominences (Figure 4).

### Wnt/β-catenin signaling and Msx1 cooperative control of the facial development

Msx genes are downstream effectors of the Bmp pathway (Marazzi et al., 1997; Bei and Maas, 1998; Tucker et al., 1998; Hollnagel et al., 1999). Msx transcription factors also act as effectors of Wnt and Fgf pathways (Chen et al., 1996; Willert et al., 2002; Hussein et al., 2003). Despite the complex gene regulatory networks that exist among these signaling pathways we were surprised to find that the interaction between Msx1 and Wnt/β-catenin signaling in the developing face is remarkably specific. The over-expression of *Wnt2b*, which exacerbates the normal palatal clefting in chick embryos, did not perturb the expression domains of three key regulators of craniofacial development: Shh, Pax6, and Fgf8.

There is undeniably a complex feedback regulation loop between Wnt/β-catenin signaling and Msx1. While our data demonstrate that Msx1 is a direct downstream target of Wnt/β-catenin signaling during craniofacial development [Figures 5, 6A and see (Song et al., 2009; Jin et al., 2011)] there is also data demonstrating that Msx1 regulates expression and activity of the Wnt/β-catenin pathway (Bach et al., 2003; Monsoro-Burq et al., 2005; Revet et al., 2010; Nallasamy et al., 2012). Clearly, a better understanding of this feedback loop will help unravel the initiating events that lead to CP. During later growth (Coudert et al., 2005), the bidirectional transcription of Msx1 homeobox gene would additionally intervene by finely controlling cell protein levels (Petit et al., 2009).

### Palatal clefting results from an imbalance in Msx1 activity

Teratologists and geneticists recognize that both excesses and deficiencies in proteins can lead to similar phenotypes. For example, mutations that causes abrogation of the Fgf receptor 2 (Fgfr2) cause hypertelorism, craniosynostosis, and mid-facial hypoplasia (Hajihosseini et al., 2001; Eswarakumar et al., 2002). Gain of function mutations that lead to constitutively active Fgfr2 cause very similar phenotypes (Chen et al., 2003; Eswarakumar et al., 2004; Wang et al., 2005; Yin et al., 2008; Holmes et al., 2009). Likewise, a reduction in (Helms et al., 1996) or an excess of (Schneider et al., 1999) retinoic acid cause very similar truncations in facial outgrowth.

By using both loss- and gain-of-function approaches in two different species we demonstrated the conserved role of Msx1 in regulating outgrowth of the maxillary prominences. We showed that the lack and ectopic expression of the same protein caused equivalent phenotypes in embryo facial development: shortened rostrum/ upper beak, and cleft palate. These data strongly suggest that there exist precise molecular mechanisms regulating the transcriptional repressor activity of Msx1 during embryogenesis since disturbance of this balance in either direction leads to maxillary growth disturbances.

In conclusion, we show that E12.5 is a critical stage in facial development for Msx1 function, and that imbalances in Wnt signaling and Msx1 activity lead to failures in craniofacial development that ultimately manifest as cleft palate.

### Conflict of interest statement

The authors declare that the research was conducted in the absence of any commercial or financial relationships that could be construed as a potential conflict of interest.

## References

[B1] Abzhanov A. Cordero D. R. Sen J. Tabin C. J. Helms J. A. (2007). Cross-regulatory interactions between Fgf8 and Shh in the avian frontonasal prominence. Congenit. Anom. (Kyoto) 47, 136–148 10.1111/j.1741-4520.2007.00162.x 17988255

[B2] Alappat S. Zhang Z. Y. Chen Y. P. (2003). Msx homeobox gene family and craniofacial development. Cell Res. 13, 429–442 10.1038/sj.cr.7290185 14728799

[B3] Albelda S. M. Muller W. A. Buck C. A. Newman P. J. (1991). Molecular and cellular properties of PECAM-1 (endoCAM/CD31): a novel vascular cell-cell adhesion molecule. J. Cell. Biol. 114, 1059–1068 1874786 10.1083/jcb.114.5.1059 PMC2289123

[B4] Albrecht U. E. G. Helms J. A. Lin H. (1997). Visualization of gene expression patterns by *in situ* hybridization, in Molecular and Cellular Methods in Developmental Toxicology, ed Daston G. P. (Boca Raton, FL: CRC Press), 23–48

[B5] Bach A. Lallemand Y. Nicola M. A. Ramos C. Mathis L. Maufras M. Robert B. (2003). Msx1 is required for dorsal diencephalon patterning. Development 130, 4025–4036 10.1242/dev.00609 12874124

[B6] Bei M. Maas R. (1998). FGFs and BMP4 induce both Msx1-independent and Msx1-dependent signaling pathways in early tooth development. Development 125, 4325–4333 9753686 10.1242/dev.125.21.4325

[B7] Bell E. J. Brickell P. M. (1997). Replication-competent retroviral vectors for expressing genes in avian cells *in vitro* and *in vivo*. Mol. Biotechnol. 7, 289–298 10.1007/BF02740819 9219242

[B8] Boughner J. C. Hallgrimsson B. (2008). Biological spacetime and the temporal integration of functional modules: a case study of dento-gnathic developmental timing. Dev. Dyn. 237, 1–17 10.1002/dvdy.21383 18058914

[B9] Boughner J. C. Wat S. Diewert V. M. Young N. M. Browder L. W. Hallgrimsson B. (2008). Short-faced mice and developmental interactions between the brain and the face. J. Anat. 213, 646–662 10.1111/j.1469-7580.2008.00999.x 19094181 PMC2666134

[B10] Brugmann S. A. Kim J. Helms J. A. (2006). Looking different: understanding diversity in facial form. Am. J. Med. Genet. A 140, 2521–2529 10.1002/ajmg.a.31361 16838331

[B11] Brugmann S. A. Moody S. A. (2005). Induction and specification of the vertebrate ectodermal placodes: precursors of the cranial sensory organs. Biol. Cell. 97, 303–319 10.1042/BC20040515 15836430

[B12] Brugmann S. A. Powder K. E. Young N. M. Goodnough L. H. Hahn S. M. James A. W. Helms J. A. Lovett M. (2010). Comparative gene expression analysis of avian embryonic facial structures reveals new candidates for human craniofacial disorders. Hum. Mol. Genet. 19, 920–930 10.1093/hmg/ddp559 20015954 PMC2816616

[B13] Chen L. Li D. Li C. Engel A. Deng C. X. (2003). A Ser252Trp [corrected] substitution in mouse fibroblast growth factor receptor 2 (Fgfr2) results in craniosynostosis. Bone 33, 169–178 10.1016/S8756-3282(03)00222-9 14499350

[B14] Chen Y. Bei M. Woo I. Satokata I. Maas R. (1996). Msx1 controls inductive signaling in mammalian tooth morphogenesis. Development 122, 3035–3044 8898217 10.1242/dev.122.10.3035

[B15] Cho H. H. Kim Y. J. Kim S. J. Kim J. H. Bae Y. C. Ba B. Jung J. S. (2006). Endogenous Wnt signaling promotes proliferation and suppresses osteogenic differentiation in human adipose derived stromal cells. Tissue Eng. 12, 111–121 10.1089/ten.2006.12.111 16499448

[B16] Cho S. H. Cepko C. L. (2006). Wnt2b/beta-catenin-mediated canonical Wnt signaling determines the peripheral fates of the chick eye. Development 133, 3167–3177 10.1242/dev.02474 16854977

[B17] Cordero D. Marcucio R. Hu D. Gaffield W. Tapadia M. Helms J. A. (2004). Temporal perturbations in sonic hedgehog signaling elicit the spectrum of holoprosencephaly phenotypes. J. Clin. Invest. 114, 485–494 10.1172/JCI19596 15314685 PMC506789

[B18] Coudert A. E. Pibouin L. Vi-Fane B. Thomas B. L. Macdougall M. Choudhury A. Robert B. Sharpe P. T. Berdal A. Lezot F. (2005). Expression and regulation of the Msx1 natural antisense transcript during development. Nucleic Acids Res. 33, 5208–5218 10.1093/nar/gki831 16157866 PMC1214550

[B19] Dixon M. J. Marazita M. L. Beaty T. H. Murray J. C. (2011). Cleft lip and palate: understanding genetic and environmental influences. Nat. Rev. Genet. 12, 167–178 10.1038/nrg2933 21331089 PMC3086810

[B20] Eswarakumar V. P. Horowitz M. C. Locklin R. Morriss-Kay G. M. Lonai P. (2004). A gain-of-function mutation of Fgfr2c demonstrates the roles of this receptor variant in osteogenesis. Proc. Natl. Acad. Sci. U.S.A. 101, 12555–12560 10.1073/pnas.0405031101 15316116 PMC515096

[B21] Eswarakumar V. P. Monsonego-Ornan E. Pines M. Antonopoulou I. Morriss-Kay G. M. Lonai P. (2002). The IIIc alternative of Fgfr2 is a positive regulator of bone formation. Development 129, 3783–3793 12135917 10.1242/dev.129.16.3783

[B22] Goudreau G. Petrou P. Reneker L. W. Graw J. Loster J. Gruss P. (2002). Mutually regulated expression of Pax6 and Six3 and its implications for the Pax6 haploinsufficient lens phenotype. Proc. Natl. Acad. Sci. U.S.A. 99, 8719–8724 10.1073/pnas.132195699 12072567 PMC124365

[B23] Hajihosseini M. K. Wilson S. De Moerlooze L. Dickson C. (2001). A splicing switch and gain-of-function mutation in FgfR2-IIIc hemizygotes causes Apert/Pfeiffer-syndrome-like phenotypes. Proc. Natl. Acad. Sci. U.S.A. 98, 3855–3860 10.1073/pnas.071586898 11274405 PMC31142

[B24] Han J. Ito Y. Yeo J. Y. Sucov H. M. Maas R. Chai Y. (2003). Cranial neural crest-derived mesenchymal proliferation is regulated by Msx1-mediated p19(INK4d) expression during odontogenesis. Dev. Biol. 261, 183–196 10.1016/S0012-1606(03)00300-2 12941628

[B25] Helms J. A. Kim C. H. Eichele G. Thaller C. (1996). Retinoic acid signaling is required during early chick limb development. Development 122, 1385–1394 8625827 10.1242/dev.122.5.1385

[B26] Helms J. A. Schneider R. A. (2003). Cranial skeletal biology. Nature 423, 326–331 10.1038/nature01656 12748650

[B27] Hilliard S. A. Yu L. Gu S. Zhang Z. Chen Y. P. (2005). Regional regulation of palatal growth and patterning along the anterior-posterior axis in mice. J. Anat. 207, 655–667 10.1111/j.1469-7580.2005.00474.x 16313398 PMC1571556

[B28] Hollnagel A. Oehlmann V. Heymer J. Ruther U. Nordheim A. (1999). Id genes are direct targets of bone morphogenetic protein induction in embryonic stem cells. J. Biol. Chem. 274, 19838–19845 10.1074/jbc.274.28.19838 10391928

[B29] Holmes G. Rothschild G. Roy U. B. Deng C. X. Mansukhani A. Basilico C. (2009). Early onset of craniosynostosis in an Apert mouse model reveals critical features of this pathology. Dev. Biol. 328, 273–284 10.1016/j.ydbio.2009.01.026 19389359 PMC2674120

[B30] Houzelstein D. Cohen A. Buckingham M. E. Robert B. (1997). Insertional mutation of the mouse *Msx1* homeobox gene by an nlacZ reporter gene. Mech. Dev. 65, 123–133 9256350 10.1016/s0925-4773(97)00065-8

[B31] Hu G. Lee H. Price S. M. Shen M. M. Abate-Shen C. (2001). Msx homeobox genes inhibit differentiation through upregulation of cyclin D1. Development 128, 2373–2384 11493556 10.1242/dev.128.12.2373

[B32] Hussein S. M. Duff E. K. Sirard C. (2003). Smad4 and beta-catenin co-activators functionally interact with lymphoid-enhancing factor to regulate graded expression of Msx2. J. Biol. Chem. 278, 48805–48814 10.1074/jbc.M305472200 14551209

[B33] Ishii M. Han J. Yen H. Y. Sucov H. M. Chai Y. Maxson R. E. Jr. (2005). Combined deficiencies of Msx1 and Msx2 cause impaired patterning and survival of the cranial neural crest. Development 132, 4937–4950 10.1242/dev.02072 16221730

[B34] Jeon H. Ono M. Kumagai C. Miki K. Morita A. Kitagawa Y. (1996). Pericytes from microvessel fragment produce type IV collagen and multiple laminin isoforms. Biosci. Biotechnol. Biochem. 60, 856–861 8704315 10.1271/bbb.60.856

[B35] Jezewski P. A. Vieira A. R. Nishimura C. Ludwig B. Johnson M. O'Brien S. E. Daack-Hirsch S. Schultz R. E. Weber A. Nepomucena B. Romitti P. A. Christensen K. Orioli I. M. Castilla E. E. Machida J. Natsume N. Murray J. C. (2003). Complete sequencing shows a role for MSX1 in non-syndromic cleft lip and palate. J. Med. Genet. 40, 399–407 10.1136/jmg.40.6.399 12807959 PMC1735501

[B36] Jin Y. R. Turcotte T. J. Crocker A. L. Han X. H. Yoon J. K. (2011). The canonical Wnt signaling activator, R-spondin2, regulates craniofacial patterning and morphogenesis within the branchial arch through ectodermal-mesenchymal interaction. Dev. Biol. 352, 1–13 10.1016/j.ydbio.2011.01.004 21237142 PMC3089906

[B37] Juriloff D. M. Harris M. J. (2008). Mouse genetic models of cleft lip with or without cleft palate. Birth Defects Res. A Clin. Mol. Teratol. 82, 63–77 10.1002/bdra.20430 18181213

[B38] Juriloff D. M. Harris M. J. Dewell S. L. Brown C. J. Mager D. L. Gagnier L. Mah D. G. (2005). Investigations of the genomic region that contains the clf1 mutation, a causal gene in multifactorial cleft lip and palate in mice. Birth Defects Res. A Clin. Mol. Teratol. 73, 103–113 10.1002/bdra.20106 15690355

[B39] Juriloff D. M. Harris M. J. McMahon A. P. Carroll T. J. Lidral A. C. (2006). Wnt9b is the mutated gene involved in multifactorial nonsyndromic cleft lip with or without cleft palate in A/WySn mice, as confirmed by a genetic complementation test. Birth Defects Res. A Clin. Mol. Teratol. 76, 574–579 10.1002/bdra.20302 16998816

[B40] Levi G. Mantero S. Barbieri O. Cantatore D. Paleari L. Beverdam A. Genova F. Robert B. Merlo G. R. (2006). Msx1 and Dlx5 act independently in development of craniofacial skeleton, but converge on the regulation of Bmp signaling in palate formation. Mech. Dev. 123, 3–16 10.1016/j.mod.2005.10.007 16330189

[B41] Lidral A. C. Romitti P. A. Basart A. M. Doetschman T. Leysens N. J. Daack-Hirsch S. Semina E. V. Johnson L. R. Machida J. Burds A. Parnell T. J. Rubenstein J. L. Murray J. C. (1998). Association of MSX1 and TGFB3 with nonsyndromic clefting in humans. Am. J. Hum. Genet. 63, 557–568 10.1086/301956 9683588 PMC1377298

[B42] Lopes M. Goupille O. Saint Cloment C. Lallemand Y. Cumano A. Robert B. (2011). Msx genes define a population of mural cell precursors required for head blood vessel maturation. Development 138, 3055–3066 10.1242/dev.063214 21693521

[B43] Macdonald R. Barth K. A. Xu Q. Holder N. Mikkola I. Wilson S. W. (1995). Midline signalling is required for Pax gene regulation and patterning of the eyes. Development 121, 3267–3278 7588061 10.1242/dev.121.10.3267

[B44] Mackenzie A. Leeming G. L. Jowett A. K. Ferguson M. W. Sharpe P. T. (1991). The homeobox gene Hox 7.1 has specific regional and temporal expression patterns during early murine craniofacial embryogenesis, especially tooth development *in vivo* and *in vitro*. Development 111, 269–285 1680043 10.1242/dev.111.2.269

[B45] Marazzi G. Wang Y. Sassoon D. (1997). Msx2 is a transcriptional regulator in the BMP4-mediated programmed cell death pathway. Dev. Biol. 186, 127–138 10.1006/dbio.1997.8576 9205134

[B46] Marcucio R. S. Cordero D. R. Hu D. Helms J. A. (2005). Molecular interactions coordinating the development of the forebrain and face. Dev. Biol. 284, 48–61 10.1016/j.ydbio.2005.04.030 15979605

[B47] Monsoro-Burq A. H. Wang E. Harland R. (2005). Msx1 and Pax3 cooperate to mediate FGF8 and WNT signals during Xenopus neural crest induction. Dev. Cell 8, 167–178 10.1016/j.devcel.2004.12.017 15691759

[B48] Nallasamy S. Li Q. Bagchi M. K. Bagchi I. C. (2012). Msx homeobox genes critically regulate embryo implantation by controlling paracrine signaling between uterine stroma and epithelium. PLoS Genet. 8:e1002500 10.1371/journal.pgen.1002500 22383889 PMC3285581

[B49] Odelberg S. J. Kollhoff A. Keating M. T. (2000). Dedifferentiation of mammalian myotubes induced by msx1. Cell 103, 1099–1109 10.1016/S0092-8674(00)00212-9 11163185

[B50] Petit S. Meary F. Pibouin L. Jeanny J. C. Fernandes I. Poliard A. Hotton D. Berdal A. Babajko S. (2009). Autoregulatory loop of Msx1 expression involving its antisense transcripts. J. Cell. Physiol. 220, 303–310 10.1002/jcp.21762 19334036

[B51] Ramos C. Fernandez-Llebrez P. Bach A. Robert B. Soriano E. (2004). Msx1 disruption leads to diencephalon defects and hydrocephalus. Dev. Dyn. 230, 446–460 10.1002/dvdy.20070 15188430

[B52] Revet I. Huizenga G. Koster J. Volckmann R. van Sluis P. Versteeg R. Geerts D. (2010). MSX1 induces the Wnt pathway antagonist genes DKK1, DKK2, DKK3, and SFRP1 in neuroblastoma cells, but does not block Wnt3 and Wnt5A signalling to DVL3. Cancer Lett. 289, 195–207 10.1016/j.neuint.2009.07.003 19815336

[B53] Salahshourifar I. Halim A. S. Wan Sulaiman W. A. Zilfalil B. A. (2011). Contribution of MSX1 variants to the risk of non-syndromic cleft lip and palate in a Malay population. J. Hum. Genet. 56, 755–758 10.1038/jhg.2011.95 21866112

[B54] Satokata I. Maas R. (1994). Msx1 deficient mice exhibit cleft palate and abnormalities of craniofacial and tooth development. Nat. Genet. 6, 348–356 7914451 10.1038/ng0494-348

[B55] Schneider R. A. Helms J. A. (2003). The cellular and molecular origins of beak morphology. Science 299, 565–568 10.1126/science.1077827 12543976

[B56] Schneider R. A. Hu D. Helms J. A. (1999). From head to toe: conservation of molecular signals regulating limb and craniofacial morphogenesis. Cell Tissue Res. 296, 103–109 10.1007/s004410051271 10199970

[B57] Schneider R. A. Hu D. Rubenstein J. L. Maden M. Helms J. A. (2001). Local retinoid signaling coordinates forebrain and facial morphogenesis by maintaining FGF8 and SHH. Development 128, 2755–2767 11526081 10.1242/dev.128.14.2755

[B58] Song L. Li Y. Wang K. Wang Y. Z. Molotkov A. Gao L. Zhao T. Yamagami T. Wang Y. Gan Q. Pleasure D. E. Zhou C. J. (2009). Lrp6-mediated canonical Wnt signaling is required for lip formation and fusion. Development 136, 3161–3171 10.1242/dev.037440 19700620

[B59] Suazo J. Santos J. L. Jara L. Blanco R. (2010). Parent-of-origin effects for MSX1 in a Chilean population with nonsyndromic cleft lip/palate. Am. J. Med. Genet. A 152A, 2011–2016 10.1002/ajmg.a.33528 20635363

[B60] Suzuki Y. Jezewski P. A. Machida J. Watanabe Y. Shi M. Cooper M. E. Viet le T. Nguyen T. D. Hai H. Natsume N. Shimozato K. Marazita M. L. Murray J. C. (2004). In a Vietnamese population, MSX1 variants contribute to cleft lip and palate. Genet. Med. 6, 117–125 15354328 10.1097/01.gim.0000127275.52925.05

[B61] Tongkobpetch S. Siriwan P. Shotelersuk V. (2006). MSX1 mutations contribute to nonsyndromic cleft lip in a Thai population. J. Hum. Genet. 51, 671–676 10.1007/s10038-006-0006-4 16868654

[B62] Tucker A. S. Al Khamis A. Sharpe P. T. (1998). Interactions between Bmp-4 and Msx-1 act to restrict gene expression to odontogenic mesenchyme. Dev. Dyn. 212, 533–539 10.1002/(SICI)1097-0177(199808)212:4<533::AID-AJA6>3.0.CO;2-I 9707326

[B63] van Amerongen R. Fuerer C. Mizutani M. Nusse R. (2012). Wnt5a can both activate and repress Wnt/beta-catenin signaling during mouse embryonic development. Dev. Biol. 369, 101–114 10.1016/j.ydbio.2012.06.020 22771246 PMC3435145

[B64] Vieira A. R. Orioli I. M. Castilla E. E. Cooper M. E. Marazita M. L. Murray J. C. (2003). MSX1 and TGFB3 contribute to clefting in South America. J. Dent. Res. 82, 289–292 10.1177/154405910308200409 12651933

[B65] Wang S. Yu X. Zhang T. Zhang X. Zhang Z. Chen Y. (2004). Chick Pcl2 regulates the left-right asymmetry by repressing Shh expression in Hensen's node. Development 131, 4381–4391 10.1242/dev.01269 15294861

[B66] Wang Y. Xiao R. Yang F. Karim B. O. Iacovelli A. J. Cai J. Lerner C. P. Richtsmeier J. T. Leszl J. M. Hill C. A. Yu K. Ornitz D. M. Elisseeff J. Huso D. L. Jabs E. W. (2005). Abnormalities in cartilage and bone development in the Apert syndrome FGFR2 +/S22W mouse. Development 132, 3537–3548 10.1242/dev.01914 15975938

[B67] Willert J. Epping M. Pollack J. R. Brown P. O. Nusse R. (2002). A transcriptional response to Wnt protein in human embryonic carcinoma cells. BMC Dev. Biol. 2, 8 10.1186/1471-213X-2-8 12095419 PMC117803

[B68] Yin L. Du X. Li C. Xu X. Chen Z. Su N. Zhao L. Qi H. Li F. Xue J. Yang J. Jin M. Deng C. Chen L. (2008). A Pro253Arg mutation in fibroblast growth factor receptor 2 (Fgfr2) causes skeleton malformation mimicking human Apert syndrome by affecting both chondrogenesis and osteogenesis. Bone 42, 631–643 10.1016/j.bone.2007.11.019 18242159

[B69] Zhang Z. Song Y. Zhao X. Zhang X. Fermin C. Chen Y. (2002). Rescue of cleft palate in Msx1-deficient mice by transgenic Bmp4 reveals a network of BMP and Shh signaling in the regulation of mammalian palatogenesis. Development 129, 4135–4146 12163415 10.1242/dev.129.17.4135

